# Supplementary material for: hsa_circ_0003738 Inhibits the Suppressive Function of Tregs by Targeting miR-562/IL-17A and miR-490-5p/IFN-γ Signaling Pathway
Source: Mol Ther Nucleic Acids. 2020 Aug 5;21:1111–9. doi: 10.1016/j.omtn.2020.08.001 (PMC7475646; doi:10.1016/j.omtn.2020.08.001)
Supplement: Document S1. Tables S1–S3 [file mmc1.pdf]

OMTN, Volume *21*

Supplemental Information

hsa_circ_0003738 Inhibits the Suppressive Function of Tregs by Targeting miR-562/IL-17A and miR-490-5p/IFN-γ Signaling Pathway

Luting Yang, Chen Zhang, Xiaocui Bai, Chunying Xiao, Erle Dang, and Gang Wang

Supplementary Materials and Methods

Circular RNA hsa_circ_0003738 inhibits the suppressive function of Tregs in psoriatic patients by targeting miR-562/IL-17A and miR-490-5p/IFN-γ signaling pathway

Table S1 Upregulated circRNAs in psoriatic Tregs

**P-value**	**FC (abs)**	**circRNA**	**Alias**	**circRNA_type**
0.034084828	3.3505229	hsa_circRNA_001175	hsa_circ_0001658	exonic
0.030387321	1.5121903	hsa_circRNA_100119	hsa_circ_0002909	exonic
0.032145082	1.5124685	hsa_circRNA_100120	hsa_circ_0000038	exonic
0.040528595	1.7819617	hsa_circRNA_100750	hsa_circ_0020929	exonic
0.04883818	1.6535833	hsa_circRNA_101031	hsa_circ_0007478	exonic
0.044809406	1.547614	hsa_circRNA_101141	hsa_circ_0005785	exonic
0.0458853	1.5419384	hsa_circRNA_101550	hsa_circ_0035796	exonic
0.032128144	1.5279761	hsa_circRNA_101740	hsa_circ_0008616	exonic
0.049057817	2.3222936	hsa_circRNA_101748	hsa_circ_0003645	exonic
0.01810066	2.1755129	hsa_circRNA_101838	hsa_circ_0039930	exonic
0.005943973	1.5254679	hsa_circRNA_103417	hsa_circ_0004788	exonic
0.036592273	1.7508295	hsa_circRNA_103572	hsa_circ_0008351	exonic
0.005949669	4.6603878	hsa_circRNA_104086	hsa_circ_0003738	exonic
0.045574811	1.79207	hsa_circRNA_104499	hsa_circ_0082564	exonic
0.005987429	1.6234627	hsa_circRNA_104940	hsa_circ_0089153	exonic
0.022270222	1.7316345	hsa_circRNA_400091	hsa_circ_0092310	intronic

Table S2 Downregulated circRNAs in psoriatic Tregs

**P-value**	**FC (abs)**	**circRNA**	**Alias**	**circRNA_type**
0.03977296	3.054981	hsa_circRNA_000167	hsa_circ_0000518	intragenic
0.043291906	1.9914322	hsa_circRNA_000274	hsa_circ_0000919	intronic
0.043480055	2.0152613	hsa_circRNA_001059	hsa_circ_0000554	intragenic
0.039331832	1.9960006	hsa_circRNA_001379	hsa_circ_0000516	antisense
0.049249289	2.5803992	hsa_circRNA_001678	hsa_circ_0000517	intragenic
0.043876847	3.0505549	hsa_circRNA_001846	hsa_circ_0000520	intragenic
0.013501149	1.5123283	hsa_circRNA_101592	hsa_circ_0036287	exonic

Table S3 Primers used in this study

Gene symbol		Sequences
Human IFN-γ	Forward	GAGTGTGGAGACCATCAAGGA
	Reverse	GTATTGCTTTGCGTTGGACA
Human IL-17	Forward	CGGACTGTGATGGTCAACCTGAAC
	Reverse	GGTCCTCATTGCGGTGGAGATTC
Human RORγT	Forward	AGATACCCTCACCTACACCTTG
	Reverse	CCGCTCAGGGCTGTATTCAA
Human RPLP0	Forward	CGTCCTCGTGGAAGTGACAT
	Reverse	CATTCCCCCGGATATGAGGC
Human	Forward	TGAGGTGCTGGCTCACATAG

Circ_0003738	Reverse	AACGACGCCATGAATCTCCA
Human	Forward	TCATCGAGCCCATTATGAAACTGA
Circ_00039930	Reverse	AATGAGCTGGTAAAGGCCAGT
Human	Forward	GCCCAATCTCTCCTGCAAGT
Circ_0001658	Reverse	CCACCTAGGAGGAACTGACAA
