# Supplementary material for: hsa_circ_0003738 Inhibits the Suppressive Function of Tregs by Targeting miR-562/IL-17A and miR-490-5p/IFN-γ Signaling Pathway
Source: Mol Ther Nucleic Acids. 2020 Aug 5;21:1111–9. doi: 10.1016/j.omtn.2020.08.001 (PMC7475646; doi:10.1016/j.omtn.2020.08.001)
Supplement: Document S2. Article plus Supplemental Information [file mmc2.pdf]

Molecular Therapy
Nucleic Acids
Original Article

hsa_circ_0003738 Inhibits the Suppressive Function of Tregs by Targeting miR-562/IL-17A and miR-490-5p/IFN-γ Signaling Pathway

Luting Yang,[1,2,3] Chen Zhang,[1,3] Xiaocui Bai,[1,3] Chunying Xiao,[1] Erle Dang,[1] and Gang Wang[1]

[1]Department of Dermatology, Xijing Hospital, Fourth Military Medical University, Xi'an 710032, China; [2]Laboratory of Gene Therapy, Department of Biochemistry, College of Life Sciences, Shaanxi Normal University, No. 620, South Chang'an Road, Xi'an 710062, China

Dysfunction in the suppressive function of regulatory T cells (Tregs) has been related to the pathogenesis of psoriasis. Accumulating evidence has demonstrated the importance of circular RNAs (circRNAs) in regulating various biological process, such as cell proliferation, apoptosis, etc. However, the role of circRNAs in modulating the suppressive functions of psoriatic Tregs and the underlying mechanisms have not been investigated. Here, by using circRNA microarray analysis, we discovered four upregulated and four downregulated circRNAs in psoriatic Tregs. Quantitative real-time PCR further confirmed a significant increase of circ_0003738 in psoriatic Tregs. Importantly, knockdown of circ_0003738 by lentivirus in psoriatic Tregs could restore their suppressive functions via inhibiting the secretion of proinflammatory cytokines interleukin-17A (IL-17A) and interferon (IFN)-γ. Moreover, we found that circ_0003738 could bind to miR-562 to release the inhibition of target gene IL-17RA (IL-17 receptor A), thus promoting IL-17A signaling in psoriatic Tregs. In parallel, circ_0003738 acted also as a sponge for miR-490-5p and relieved inhibition for the target gene IFNGR2, which promoted IFN-γ signaling in psoriatic Tregs. Our study demonstrated that upregulated circ_0003738 decreased the suppressive function of psoriatic Tregs via the miR-562/IL17RA and miR-490-5p/IFNGR2 (IFN-γ receptor 2) axis, which indicated the involvement of circRNAs in the pathogenesis of dysfunctional Tregs. These findings will provide new therapeutic targets for the treatment of psoriasis.

INTRODUCTION

Psoriasis is a chronic inflammatory skin disease characterized by keratinocyte hyperproliferation and infiltration of immune cells in the dermis. The crosstalk among different cells, such as T cells, keratinocytes, neutrophils, macrophages, and dendritic cells, has long been considered to be involved in the pathogenesis of psoriasis. Even though the etiology of psoriasis remains unclarified, it is believed to be a multifactorial disease with key components, including genetic factors and environmental factors. For example, immunological abnormalities, including inflammatory cytokines and aberrant activation of signaling pathways, gene susceptibility loci such as the 13 identified psoriasis susceptibility locus (PSORS), and environmental factors, including stress, smoking, and infections, have all contributed to the development of psoriasis. Besides, dysregulation in the epigenetic network, especially the role of non-coding RNAs (ncRNAs), has offered pivotal pathogenic insights regarding psoriasis pathogenesis.

Circular RNAs (circRNAs), a novel member of ncRNAs, are characterized by the covalent circular structure formed by the 5′ end of one exon with the 3′ end of another.[1] circRNAs are abundant in mammals and are identified to be cell and tissue specific.[2,3] Increasing evidence has suggested that circRNAs function by acting as a sponge for microRNAs (miRNAs) and RNA binding proteins (RBPs), which regulate the gene expression at the transcriptional or post-transcriptional level.[4] Recently, due to the development of technology in bioinformatics, circRNAs illuminate critical functions in regulating cell cycle, inflammation, cell proliferation, and metabolic process.[5] Moreover, accumulating evidence has revealed the involvement of circRNAs in the pathology of autoimmune diseases,[6] cancers,[7] cardiovascular diseases,[8,9] and neurological diseases.[10] However, only limited studies have been conducted for clarifying the role of circRNAs in psoriasis. One study indicated that hsa_circ_0061012 was upregulated in psoriatic lesions.[11] Nevertheless, the precise molecular mechanism by which circRNAs might participate in the etiology of psoriasis warrants further elucidation.

Regulatory T cells (Tregs) are a subset of immunosuppressive $CD4^+$ T cells that have dominant roles in mediating immune tolerance and maintaining immune homeostasis.[12] Deficiencies in both the number and suppressive function of Tregs are related to the pathogenesis of various autoimmune diseases, represented by psoriasis, multiple sclerosis, and systemic lupus erythematosus.[13–16] In psoriasis patients, the impaired suppressive function of Tregs led to the

Received 31 May 2020; accepted 31 July 2020;
https://doi.org/10.1016/j.omtn.2020.08.001.

[3]These authors contributed equally to this work.

Correspondence: Gang Wang, MD, PhD, Department of Dermatology, Xijing Hospital, Fourth Military Medical University, 127 Changle West Road, Xi'an 710032, China.
E-mail: xjwgang@fmmu.edu.cn

This is an open access article under the CC BY license (http://creativecommons.org/licenses/by/4.0/).

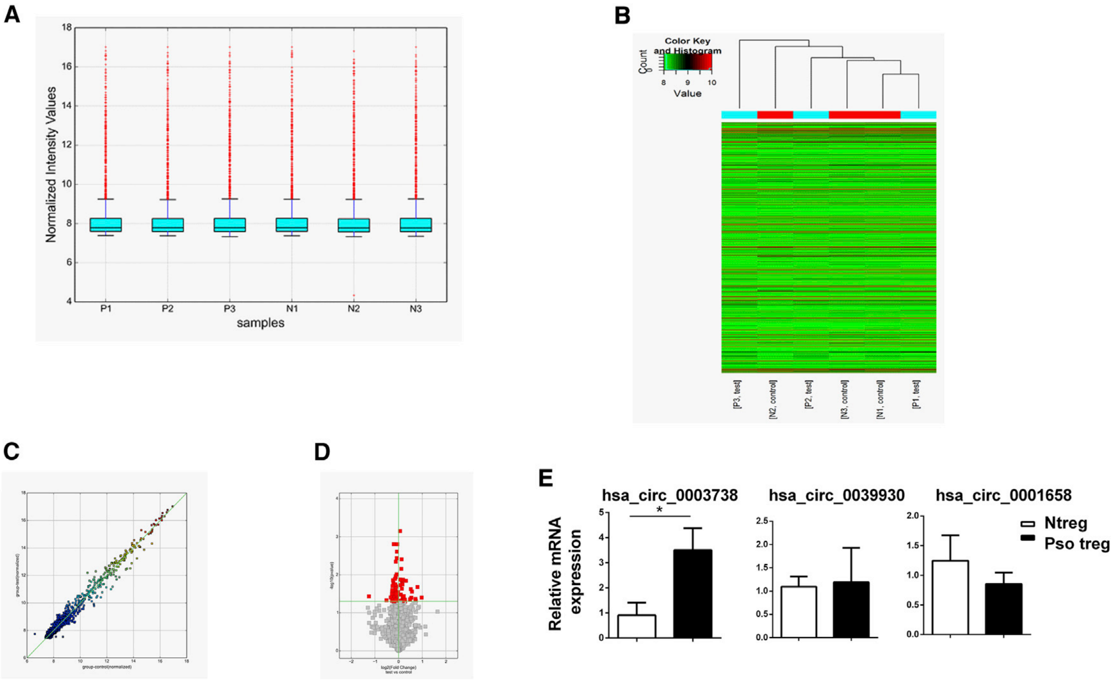

Figure 1. circ_0003738 Was Upregulated in Psoriatic Tregs

(A) Boxplots were applied to visualize the distributions of circRNAs from the six samples. (B) Heatmap showed differentially expressed circRNAs between the two groups. Red spot stands for upregulation, and green spot stands for downregulation. (C) Scatterplot showed the variations of circRNA expressions in psoriatic Tregs (y axis) and normal Tregs (x axis). The values are the averaged normalized signal values of each group (log2 scaled). (D) Volcano plot of differentially expressed circRNAs. (E) Quantitative real-time PCR analysis of circRNAs expression in Tregs from psoriatic patients (n = 8) and normal controls (n = 6). Data are representative of means ± SEMs. *p < 0.05.

uncontrolled proliferation of effector T cells (Teffs), which disturbs the immune homeostasis and contributes to the development of psoriasis. The mechanisms underlying the dysfunctional Tregs in psoriasis are multiple and encompass the aberrant activation of transcriptional factors, the high level of proinflammatory cytokines, and other mediators, such as miRNAs. Previously, we have found that aberrant activation of the STAT3 pathway because of the stimulation with interleukin (IL)-6, IL-21, and IL-23 could reverse the suppressive function of psoriatic Tregs.[17] In addition, dysregulation of protein kinase B (PKB), which induced the nuclear exclusion of Foxo1 and decreased its transcriptional activity, represented another novel mechanism for the impaired suppressive function of Tregs in psoriasis.[18] miRNAs, an important epigenetic modification, were recently uncovered to be involved in dysfunctional Tregs in psoriasis. miR-210, which is upregulated in psoriatic Tregs, could target and inhibit the expression of Foxp3 and impair the immunosuppressive functions of Tregs. However, whether circRNAs, which have gained great interest in autoimmunity because of their multi-functions, are associated with the reduced suppressive function of Tregs in psoriasis still needs further investigation.

In this study, we analyzed the expression profile of circRNAs in psoriatic Tregs in comparison with normal Tregs and identified circ_0003738 to be upregulated in psoriatic Tregs. Knockdown of circ_0003738 in psoriatic Tregs could partially restore their suppressive function through inhibiting the production of IL-17A and interferon (IFN)-γ. Moreover, we found the targets of circ_0003738 to be miR-562 and miR-490-5p, which targeted IL-17 receptor A (IL-17RA) and IFN-γ receptor 2 (IFNGR2), respectively. Our findings established a novel role of circRNAs in the regulation of Treg suppressive function in psoriasis. circ_0003738 might be a potential therapeutic target in the treatment of psoriasis.

RESULTS

circ_0003738 Expression Is Significantly Upregulated in Psoriatic Tregs

To study the expression pattern of circRNAs in normal Tregs and Tregs from psoriatic patients, we performed a circular microarray analysis. The boxplot illustrated the location and variation changes among the six samples. After log2 normalization, no abnormal dispersion of the data was observed (Figure 1A). Hierarchical clustering analysis revealed the differential expression patterns of circRNAs between the psoriatic Tregs and normal Tregs (Figure 1B). The corresponding scatterplot analysis showed important changes in the patterns of circRNAs expressed in normal and psoriatic Tregs (Figure 1C). In addition, a volcano plot identified both the upregulated and downregulated circRNAs at different p values and fold changes (FCs) between the two groups (Figure 1D). Notably, four upregulated circRNAs and four downregulated circRNAs were identified in psoriatic Tregs ($p < 0.05$; FC $\geq$ 2) (Tables S1 and S2). To verify

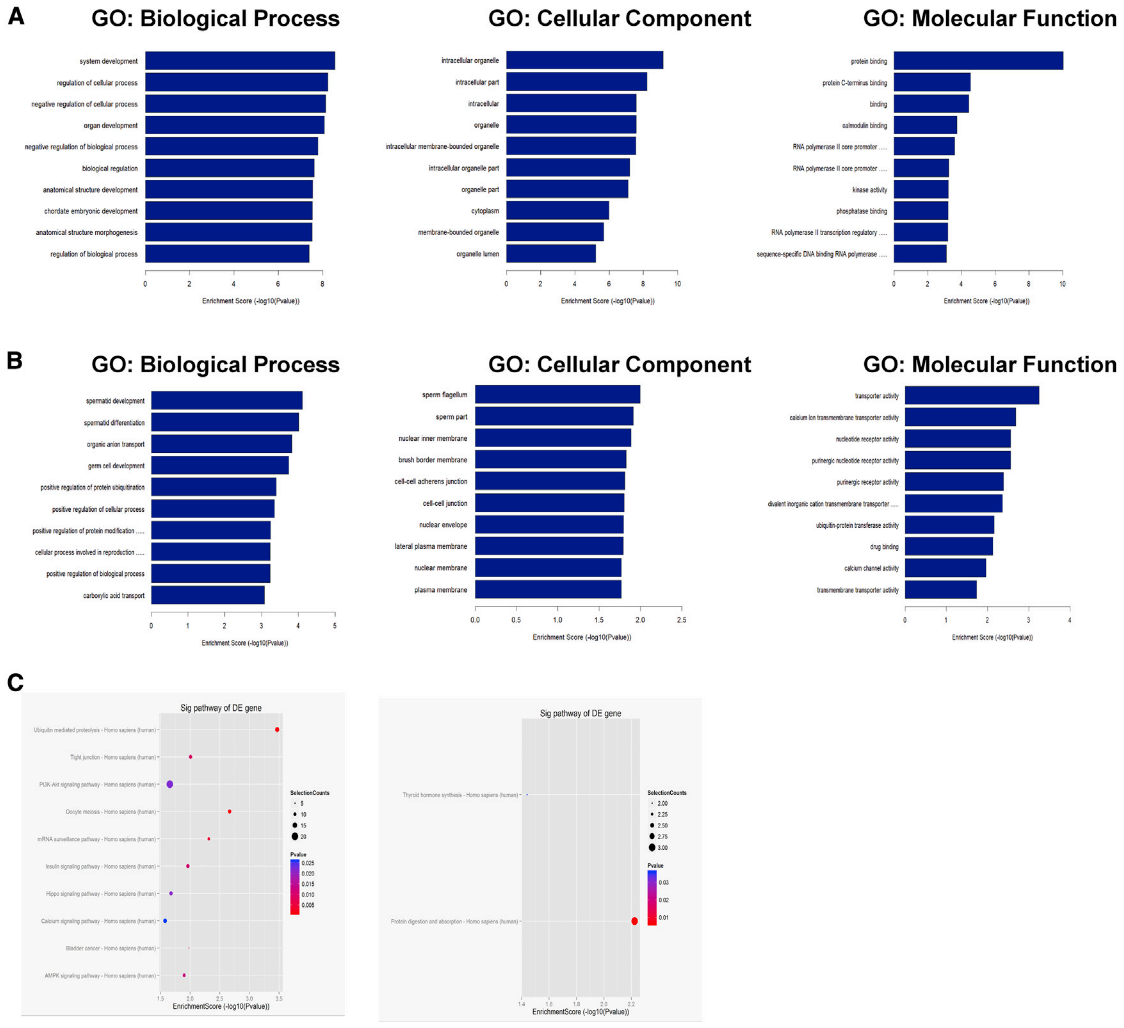

Figure 2. Gene Ontology (GO) and Kyoto Encyclopedia of Genes and Genomes (KEGG) Analysis

(A) Top 10 GO enriched biological process, cellular component, and molecular function terms for upregulated circRNA host genes. (B) Top 10 GO enriched biological process, cellular component, and molecular function terms for downregulated circRNA host genes. (C) Enrichment scores of upregulated (left) and downregulated (right) circRNA host genes according to pathway analysis. The dot plot shows the top 10 enrichment scores [−log10(p value)] of the significantly enriched pathways.

the relative expression of these differentially expressed genes, we performed quantitative real-time PCR in six samples from normal Tregs and eight samples from psoriatic Tregs. Interestingly, the top upregulated circRNA circ_0003738 with a FC of 4.66 showed a significant increase in psoriatic Tregs (Figure 1E). Thus, the role of circ_0003738 in regulating the function of psoriatic Tregs was further investigated in our study.

GO and KEGG Pathway Analysis of the Differentially Expressed circRNA Host Genes

Subsequently, Gene Ontology (GO) and Kyoto Encyclopedia of Genes and Genomes (KEGG) pathway enrichment analyses were applied to predict the potential factors and signaling pathway of the altered parental genes in psoriatic Tregs. The top GO terms for the upregulated circRNA host genes were "system development" in biological process (BP), "intracellular organelle" in cellular component (CC), and "protein binding" in molecular function (MF) (Figure 2A). Additionally, GO enrichment analysis for the downregulated circRNA host genes was "spermatid development" in BP, "sperm flagellum" in CC, and "transporter activity" for MF (Figure 2B). KEGG pathway analysis of the targeted mRNAs revealed that these upregulated circRNAs might be involved in inflammatory-related pathways, such as the phosphatidylinositol 3-kinase (PI3K)-Akt signaling pathway, AMPK pathway, and ubiquitin-mediated proteolysis (Figure 2C).

Knockdown of circ_0003738 Restored the Suppressive Function of Tregs in Psoriatic Patients

We previously demonstrated that psoriatic Tregs exhibited decreased suppressive functions, which led to uncontrolled proliferation of Teffs and development of psoriasis.[17] To analyze whether circ_0003738

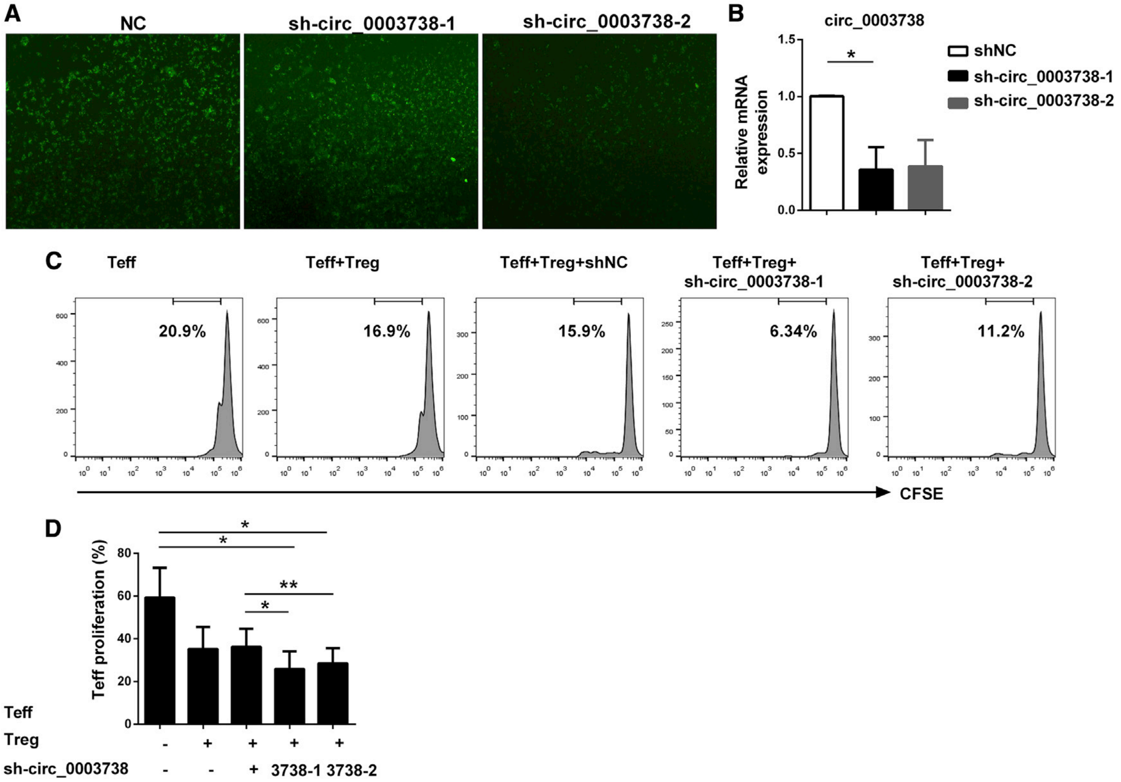

Figure 3. Knockdown of circ_0003738 Restored the Suppressive Function of Psoriatic Tregs

(A) Immunofluorescence image of psoriatic Tregs after transfection with sh-circ_0003738-1 or sh-circ_0003738-2 for 96 h. (B) Quantitative real-time PCR analysis of circ_0003738 after transfection with sh-circ_0003738. (C) Representative image showing proliferation of psoriatic Teffs cocultured with autoreactive Tregs transfected with sh-circ_0003738 for 5 days. (D) Analysis of Teff proliferation in different groups. Data are representative of means ± SEMs. *$p < 0.05$; **$p < 0.01$.

might be involved in the mechanism accounting for dysfunctional Tregs, we first knocked down the expression of cir_0003738 by lentivirus. Due to the challenge of transfection in suspended cells, we tested the transfection efficiency with sh-circ_0003738-1 and sh-circ_0003738-2 in psoriatic Tregs. Immunofluorescence showed that most of the cells could be transfected with either sh-circ_0003738-1 or sh-circ_0003738-2 (Figure 3A). Quantitative real-time PCR showed that knockdown of circ_0003738 with the two lentiviruses could reduce the expression of circ_0003738 compared with the control group (Figure 3B). More importantly, knockdown of circ_0003738 in psoriatic Tregs could significantly decrease the proliferation of Teffs, which restored the suppressive function of Tregs in psoriatic patients (Figures 3C and 3D). The above data demonstrated the promotive effect of circ_0003738 on the decreased suppressive function of Tregs in psoriasis. Knockdown of circ_0003738 could partially restore the suppressive function of psoriatic Tregs.

circ_0003738 Contributes to the Secretion of Proinflammatory Cytokine IFN-γ and IL-17A in Psoriatic Tregs

Our recent studies indicated that the dysregulation of the Th1 and Th17 program in psoriatic Tregs might be a critical factor in leading to the dysfunction of Tregs, which is evidenced by increased secretion of IFN-γ and IL-17A.[17,18] Given the evidence that circ_0003738 exerted a positive role resulting in the dysfunctional Tregs in psoriasis, we thus hypothesized that circ_0003738 might increase the secretion of these aforementioned proinflammatory cytokines in psoriatic Tregs. Flow cytometry and quantitative real-time PCR confirmed the increased expression of Th17-related cytokine IL-17A and Th1-related cytokine IFN-γ in psoriatic Tregs compared with Tregs from normal controls (Figures 4A–4C). Moreover, the transcription factor for Th17 cell differentiation named receptor-related orphan nuclear receptor γt (RORγT) was consistently upregulated in psoriatic Tregs (Figure 4C). Expectedly, the proportion of IL-17A in psoriatic $CD4^+CD25^+Foxp3^+$ Tregs was reduced from 7.71% ± 2.77% to 2.05% ± 1.42% after knockdown with sh-circ_0003738-1 (Figures 4D and 4F). Consistently, the proportion of IFN-γ in psoriatic $CD4^+CD25^+Foxp3^+$ Tregs decreased from 7.13% ± 0.71% to 3.72% ± 0.75% and 1.80% ± 1.78% after knockdown with sh-circ_0003738-1 or sh-circ_0003738-2 individually (Figures 4E and 4F). Moreover, quantitative real-time PCR supported the aforementioned results (Figure 4G). Thus, the above results suggest that aberrant upregulation of circ_0003738 decreased the suppressive function of psoriatic Tregs by promoting the secretion of Th1- and Th17-related proinflammatory cytokines IL-17A and IFN-γ.

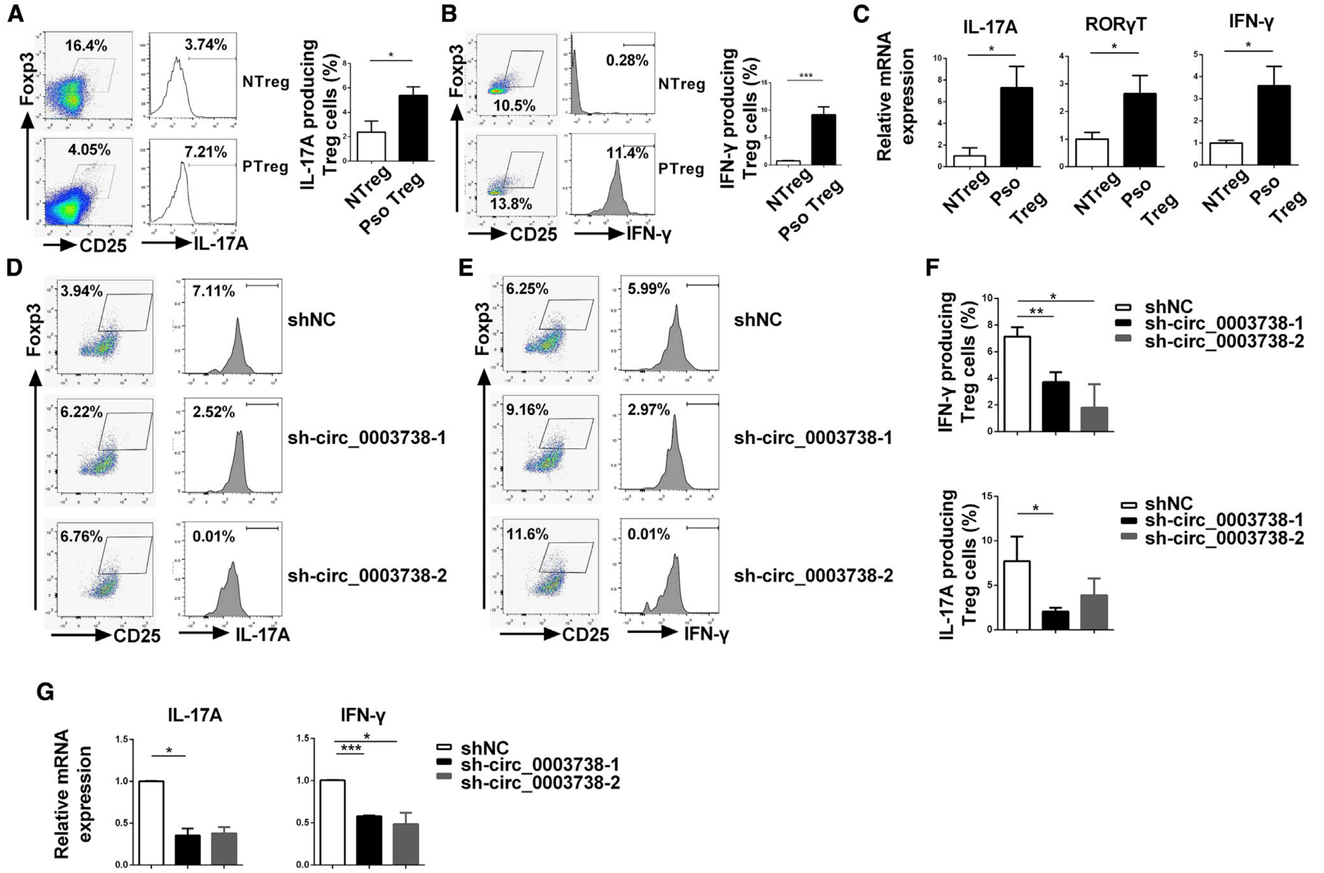

Figure 4. Knockdown of circ_0003738 in Psoriatic Tregs Suppressed the Secretion of IL-17A and IFN-γ

(A and B) Flow cytometry analysis of IL-17A (A) and IFN-γ (B) in normal Tregs and psoriatic Tregs. (C) Quantitative real-time PCR analysis of IL-17A, RORγT, and IFN-γ mRNA expression in normal and psoriatic Tregs. (D and E) Representative image of flow cytometric analysis of IL-17A (D) and IFN-γ (E) expressions after transfection with shNC or sh-circ_0003738. (F) Mean percentage of IFN-γ- (n = 4) and IL-17A-producing (n = 5) Tregs after transfection with sh-circ_0003738. (G) Quantitative real-time PCR analysis of IL-17A and IFN-γ mRNA expressions after transfection with sh-circ_0003738 in psoriatic Tregs. Data are representative of means ± SEMs. *$p < 0.05$; **$p < 0.01$; ***$p < 0.001$.

circ_0003738 Binds to miR-562 and miR-490-5p

The most important role of circRNAs is to act as the sponge to sequester miRNAs. To further explore the underlying mechanism by which circ_0003738 influenced the suppressive function in psoriatic Tregs, we performed prediction analysis based on database TargetScan and miRanda. Prediction results indicated that miR-562 and miR-490-5p might be the target of circ_0003738 in Tregs. The potential miRNA binding sites (miRNA response elements [MREs]) located in circ_0003738 were displayed in Figure 5A. Fluorescence *in situ* hybridization (FISH) was utilized to verify the binding between circ_0003738 and miR-562 and miR-490-5p, which showed that circ_0003738 colocalized with miR-562 and miR-490-5p in the cytoplasm, respectively (Figure 5B). Taken together, these results suggested that miR-562 and miR-490-5p were potential targets of circ_0003738.

circ_0003738/miR-562 Axis Upregulates IL-17A Signaling and circ_0003738/miR-490-5p Axis Upregulates IFN-γ Signaling

To clarify whether circ_0003738 could upregulate the expression of IL-17 and IFN-γ through the miR-562 and miR-490-5p axis, we determined the downstream targets of these two miRNAs by TargetScan version 6.0. After a primary selection for genes implicated in IL-17A and IFN-γ signaling, one binding site was found for miR-562 in the 3′ UTR of IL-17RA, the receptor through which IL-17A and other IL-17 family cytokines signal to exert inflammatory functions (Figure 5C). By comparison with the control group, quantitative real-time PCR showed that IL-17RA and IL-17A expressions were significantly increased by transfection with miR-562 inhibitor, whereas the expressions of IL-17RA and IL-17A were decreased after transfection with miR-562 mimics, revealing a negative modulation of miR-562 on IL-17RA and IL-17A (Figure 5D). To verify the direct binding between miR-562 and IL-17RA, we performed FISH. A co-localization between IL-17RA and miR-562 was identified mainly in the cytoplasm of Jurkat cells, showing the binding between IL-17RA and miR-562 (Figure 5E). What is more, one binding site was identified for miR-490-5p in the 3′ UTR of IFNGR2, which binds to IFNGR1 for the signaling of IFN-γ. The results from quantitative real-time PCR elucidated an increased expression of IFNGR2 and IFN-γ after miR-490-5p silencing, which was decreased after

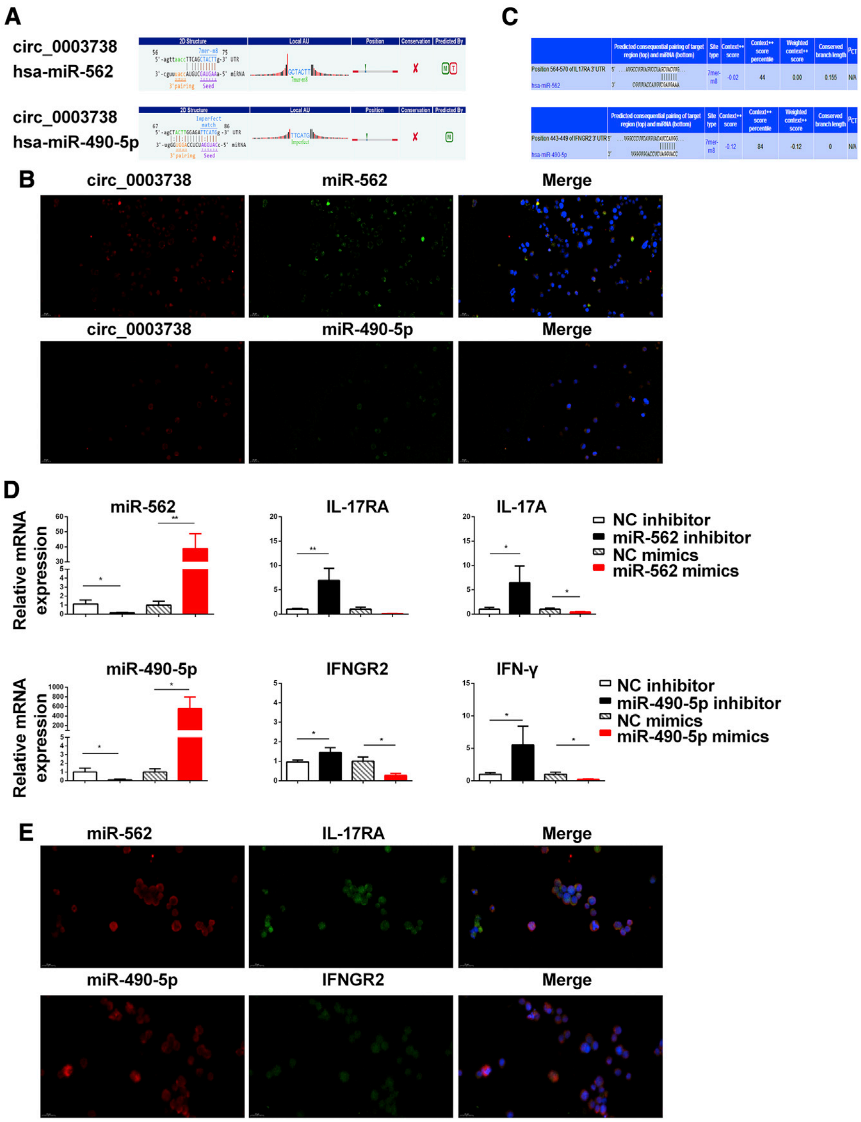

Figure 5. Identification of circ_0003738/miR-562/IL-17RA and circ_0003738/miR-490-5p/IFNGR2 Signaling

(A) TargetScan and miRanda showed that miR-562 and miR-490-5p might be the potential targets of circ_0003738. (B) FISH indicated the colocalization between circ_0003738 (red) and miR-562 (green) and miR-490-5p (green) in the cytoplasm of Jurkat cells. (C) TargetScan showing miR-562 and miR-490-5p binding sites in 3′ UTR of IL-17RA and IFNGR2. (D) Quantitative real-time PCR analysis of IL-17RA and IL-17A expression after transfection with miR-562 mimics or inhibitor. Quantitative real-time PCR analysis of IFNGR2 and IFN-γ expression after transfection with miR-490-5p inhibitor or mimics. (E) FISH showed the colocalization between miR-562 and IL-17RA, and colocalization between miR-490-5p and IFNGR2 in the cytoplasm of Jurkat cells. Data are representative of means ± SEMs. *p < 0.05; **p < 0.01.

transfection with miR-490-5p mimics (Figure 5D). FISH further confirmed the colocalization between IFNGR2 and IFN-γ in the cytoplasm of Jurkat cells (Figure 5E). In summary, these results indicated that IL-17RA is a direct target of miR-562, and IFNGR2 is a direct target of miR-490-5p. circ_0003738 could positively regulate IL-17A production and IL-17 signaling through competitively binding to miR-562 and regulate IFN-γ production and IFN-γ signaling through competitively binding to miR-490-5p.

DISCUSSION

circRNAs have been linked to the pathogenesis of various diseases. However, the role of circRNAs in the etiology of psoriasis remains largely unclarified. Two studies profiled circRNA expression in psoriatic lesional skin. In one study, nearly 5,000 differentially expressed circRNAs were identified in psoriatic lesional skin compared with normal skin tissues. By further quantitative real-time PCR analysis, upregulated circ_0061012 in psoriatic lesional skin was sorted to be the candidate biomarker.[11] In the other study, which used RNA sequencing, a substantial downregulation of circRNAs was characterized in psoriatic lesional skin compared with non-lesional skin (128 versus 285).[19] The other two studies focused on the role of circRNAs in mesenchymal stem cells (MSCs) in psoriatic lesions.[20,21] Different from other investigations, we are the first to identify the role of circRNAs in Tregs in the pathogenesis of psoriasis. In this study, we revealed that upregulated expression of has_circ_0003738 in psoriatic Tregs could impair their suppressive functions. We also found that circ_0003738 acted as the sponge for miR-562 and miR-490-5p, which released the miR-562-mediated suppression of IL-17A signaling and miR-490-5p-mediated suppression of IFN-γ signaling. Thus, the circ_0003738/miR-562 and miR-490-5p axis favors the conversion of Tregs to Th1-like and Th17-like Tregs, which might account for the decreased suppressive function of Tregs in psoriasis.

Dysfunction in the suppressive function of Tregs is critical in contribution to T cell immune imbalance and the development of psoriasis. However, the ability to characterize the mechanism accounting for dysfunctional Tregs is hindered by the limited number of Tregs in psoriasis. Proinflammatory cytokines, transcription factors, and epigenetic factors, such as miRNAs, have been pointed out to be involved. Apart from IL-6,[22] we found that other cytokines, such as IL-21 and IL-23, drove a significant activation of STAT3 signaling in psoriatic Tregs, which dampened the Treg/Teff activity.[17] We further focused on the role of transcriptional factors in regulating the function of Tregs. This led us to find that PKB induced nuclear export of Foxo1, and the subsequent loss of Foxo1 transcription activity enabled the Teff

escape from Treg-mediated suppression.[18] Besides, a critical role for miRNAs in Tregs has been discovered. For example, miR-155 could target suppressor of cytokine signaling 1 (SOCS1) to regulate Foxp3 expression and stability, which influenced Treg differentiation and development.[23,24] Other miRNAs, such as miR-146, miR-210, and miR-15a-16, have distinct roles in regulating Treg suppressive function, development, differentiation, and homeostasis via modulating the expression of different target genes.[25] In an effort to better understand other epigenetic factors involved in the mechanism underlying impaired function of Tregs in psoriasis, we focused on the novel member of ncRNAs, circRNAs. Despite the difficulty in transfection efficiency in T cells, we succeeded by using the lentivirus targeting circRNAs. Although numerous functions have elucidated the multi-functions of circRNAs in different diseases, no study has investigated the role of circRNA in Tregs. Of note, we found that upregulated expression of circ_0003738 might be another mechanism accounting for decreased suppressive function of Tregs in psoriasis. However, future studies are needed to clarify the role of other circRNAs in regulating the Treg function in disease pathogenesis.

In this study, we expand on the observation that a fraction of peripheral Tregs could convert into Th1-like and Th17-like Tregs. Not only did these cells express IL-17A, RORγT, which is the master transcription of Th17 cells, was also upregulated in psoriatic Tregs. Interestingly, this differentiation paradigm is not unique to psoriasis; other reports have pointed out this mechanism in autoimmune diseases, such as diabetes and inflammatory bowel diseases. In type 2 diabetes, the frequency of IL-17-expressing Tregs was significantly upregulated. Moreover, this type of Th17-like Treg could not express transforming growth factor β (TGF-β) and IL-10, which displayed a decreased suppressive function of Tregs.[26] In inflammatory bowel diseases, the IL-17-producing Tregs were also characterized in inflamed mucosa, which imply Th17-like Tregs are at a crossroads between Tregs and Th17 cells upon different microenvironment.[27] In asthma, the IL-4R variant directs the reprogramming of inducible Tregs (iTregs) to Th17-like cells in an IL-6-dependent manner.[28] Moreover, IL-17A^{+}Foxp3^{+} Tregs were previously described in lesions of severe psoriatic patients.[29] The inflammatory microenvironment, especially the presence of cytokines, is responsible for this programmed conversion of Tregs. Evidence is presented by recent studies that emphasize the role of IL-6, IL-23, and IL-1β.[30,31] These proinflammatory cytokines might enhance the expression of transcription regulator Id2, which reduces the expression of Foxp3 and enhances Treg plasticity. However, our work expands our current knowledge on the stimulators that drive the differentiation mode of Th1-like and Th17-like Tregs. In addition to the previously reported cytokines, such as IL-23, circ_0003738 was capable of driving the secretion of IL-17A and IFN-γ in psoriatic Tregs, which promoted the inflammatory process and disturbs the T cell homeostasis. One possibility might be that has_ circ_0003738 could promote the differentiation of Th1 and Th17 cells, resulting in the increased expression of IFN-γ and IL-17A. On the one side, TargetScan predicted transcription factors and cofactors that affect the differentiation of Th17 cells, including RORα, Runx1, and Mina, to be the target genes of miR-562. On the other side, Ets-1, a cofactor of the transcription factor of Th1 cells, T-bet, is predicted to be the potential target gene of miR-490-5p. Another possible mechanism is that miR-490-5p or miR-562, two targets of has_circ_0003738, might bind directly to the 3′ UTR of IFN-γ or IL-17A to repress their translations. This said, circRNAs, such as circ_0003738, might function through stimulating the conversion of Tregs to Th1-like and Th17-like Tregs to restrain the suppressive function of Tregs. Taken together, our work puts forward the possibility by intervention with these molecules to reduce the plasticity of these Tregs.[32]

Taken together, the findings in our paper suggest another novel mechanism by which circRNAs could regulate the impaired function of Tregs in psoriasis. This opens a new field of research for elucidation of circRNAs in the pathogenesis of dysfunctional Tregs in different autoimmune diseases. Moreover, circ_0003738, which is overexpressed in psoriatic Tregs, might be a potential therapeutic target for psoriasis treatment in future use.

MATERIALS AND METHODS

Patients and Samples

Peripheral blood was collected from three patients with moderate-to-severe psoriasis. All psoriatic patients were at progressive stage and did not receive any systemic treatment for at least 4 weeks prior to the procedures, including immunosuppressive agents or phototherapy. Blood samples from healthy controls were collected from healthy volunteers with the matched age and sex. Peripheral blood mononuclear cells (PBMCs) were prepared by Ficoll density gradient by adding lymphocyte separation media (Dakewe, Shenzhen, China). This study was approved by the Ethics Committee of Xijing Hospital. Informed consent was obtained from all individual participants included in this study.

Treg Preparation

PBMCs from healthy controls and psoriasis patients were stained with anti-CD4-phycoerythrin (PE)/Cy7 (BioLegend, CA, USA), anti-CD25-PE/Cy5 (BD Biosciences, CA, USA), and anti-CD127-allophycocyanin (APC) (BioLegend, CA, USA). CD4^{+}CD25^{+}CD127^{low} Tregs and CD4^{+}CD25^{-} Teffs were sorted by flow cytometry (Beckman Coulter, CA, USA).

circRNA Microarray Analysis

Total RNAs of sorted Tregs from three psoriatic patients and three healthy controls were extracted using RNeasy mini kit (QIAGEN, Hilden, Germany). The purity and concentration of total RNA samples were determined with NanoDrop ND-1000 (NanoDrop, DE, USA). Total RNA from each sample was treated with RNase R to enrich circRNA. The enriched circRNA was then amplified and transcribed into fluorescent cRNA utilizing random primer according to Arraystar Super RNA Labeling protocol (Arraystar, MD, USA). The labeled cRNAs were hybridized onto the Arraystar Human circRNA Arrays (8x15K; Arraystar) and incubated for 17 h at 65°C in an Agilent Hybridization Oven. After washing, slides were scanned with the Agilent Scanner G2505C (Agilent Technologies, CA, USA). Raw data were extracted, and subsequent data analysis was performed using the

R software package (provided by Kangcheng Biotechnology, Shanghai, China) (GEO: GSE154818).

Treg Suppression Assay

5-(and 6)-Carboxyfluorescein diacetate succinimidyl ester (Invitrogen, CA, USA)-stained $CD4^+CD25^-$ Teffs (5×10^4 cells) were cocultured with $CD4^+CD25^+CD127^{low}$ Tregs (5×10^4 cells) at a ratio of 1:1 in RPMI 1640 medium supplemented with 15% fetal bovine serum (FBS). Cells were cocultured in 96 U-bottom multiwell plates for 5 days. Teff proliferation was analyzed by flow cytometry based on the CFSE signal (FACSCalibur, BD, NJ, USA).

Quantitative Real-Time PCR

Total RNA was extracted using TRIzol reagent (Invitrogen, CA, USA). Quantitative real-time PCR was performed on a Chromo 4 continuous fluorescence detector with a PTC-200 DNA Engine Cycler (Bio-Rad, CA, USA). Primers are listed in Table S3.

Cell Transfection

Jurkat cells were cultured in 1640 medium supplemented with 15% FBS. Transfection with short hairpin RNA (shRNA) targeting circ_0003738 was carried out by lentiviral vectors (GenePharma, Shanghai, China). Mimics and inhibitors for miR-562 and miR-490-5p (synthesized by Ribobio Biotechnologies, Guangzhou, China) were transfected into Jurkat cells by Lipofectamine 3000 (Invitrogen, CA, USA).

FISH

Probes for circ_0003738, miR-562, miR-490-5p, IL-17RA, and IFNGR2 were synthesized by Servicebio Biotechnology (Wuhan, China). Jurkat cells were seeded into slides. The slides were baked at 62°C for 2–3 h. FISH was performed according to the manufacturer's instructions (Bosterbio, USA).

Statistical Analysis

Data between two groups were determined using Student's t tests. Comparisons of multiple groups were determined by one-way ANOVA. A p value <0.05 was considered statistically significant.

SUPPLEMENTAL INFORMATION

Supplemental Information can be found online at https://doi.org/10.1016/j.omtn.2020.08.001.

AUTHOR CONTRIBUTIONS

L.Y. designed the study and wrote the manuscript. L.Y., C.Z., and X.B. performed the experiments. C.X. and E.D. revised the manuscript and provided critical suggestions. G.W. supervised this study and revised the manuscript.

CONFLICTS OF INTEREST

The authors declare no competing interests.

ACKNOWLEDGMENTS

We thank all patients who assisted with this study. The study was supported by grants from the National Natural Science Foundation of China (81602749 and 81673051).

REFERENCES

1. Zhang, J., Chen, S., Yang, J., and Zhao, F. (2020). Accurate quantification of circular RNAs identifies extensive circular isoform switching events. Nat. Commun. *11*, 90.
2. Hsiao, K.Y., Lin, Y.C., Gupta, S.K., Chang, N., Yen, L., Sun, H.S., and Tsai, S.J. (2017). Noncoding Effects of Circular RNA CCDC66 Promote Colon Cancer Growth and Metastasis. Cancer Res. *77*, 2339–2350.
3. He, J., Huang, Z., He, M., Liao, J., Zhang, Q., Wang, S., Xie, L., Ouyang, L., Koeffler, H.P., Yin, D., and Liu, A. (2020). Circular RNA MAPK4 (circ-MAPK4) inhibits cell apoptosis via MAPK signaling pathway by sponging miR-125a-3p in gliomas. Mol. Cancer *19*, 17.
4. Li, X., Yang, L., and Chen, L.L. (2018). The Biogenesis, Functions, and Challenges of Circular RNAs. Mol. Cell *71*, 428–442.
5. Xiao, M.-S., Ai, Y., and Wilusz, J.E. (2020). Biogenesis and Functions of Circular RNAs Come into Focus. Trends Cell Biol. *30*, 226–240.
6. Xia, X., Tang, X., and Wang, S. (2019). Roles of CircRNAs in Autoimmune Diseases. Front. Immunol. *10*, 639.
7. Zhang, Q., Wang, W., Zhou, Q., Chen, C., Yuan, W., Liu, J., Li, X., and Sun, Z. (2020). Roles of circRNAs in the tumour microenvironment. Mol. Cancer *19*, 14.
8. Zhou, M.Y., Yang, J.-M., and Xiong, X.D. (2018). The emerging landscape of circular RNA in cardiovascular diseases. J. Mol. Cell. Cardiol. *122*, 134–139.
9. Aufiero, S., Reckman, Y.J., Pinto, Y.M., and Creemers, E.E. (2019). Circular RNAs open a new chapter in cardiovascular biology. Nat. Rev. Cardiol. *16*, 503–514.
10. Zhang, Y., Yu, F., Bao, S., and Sun, J. (2019). Systematic Characterization of Circular RNA-Associated CeRNA Network Identified Novel circRNA Biomarkers in Alzheimer's Disease. Front. Bioeng. Biotechnol. *7*, 222.
11. Qiao, M., Ding, J., Yan, J., Li, R., Jiao, J., and Sun, Q. (2018). Circular RNA Expression Profile and Analysis of Their Potential Function in Psoriasis. Cell. Physiol. Biochem. *50*, 15–27.
12. Deng, G., Song, X., Fujimoto, S., Piccirillo, C.A., Nagai, Y., and Greene, M.I. (2019). Foxp3 Post-translational Modifications and Treg Suppressive Activity. Front. Immunol. *10*, 2486.
13. Mexhitaj, I., Nyirenda, M.H., Li, R., O'Mahony, J., Rezk, A., Rozenberg, A., Moore, C.S., Johnson, T., Sadovnick, D., Collins, D.L., et al. (2019). Abnormal effector and regulatory T cell subsets in paediatric-onset multiple sclerosis. Brain *142*, 617–632.
14. Zhao, C., Chu, Y., Liang, Z., Zhang, B., Wang, X., Jing, X., Hao, M., Wang, Y., An, J., Zhang, X., et al. (2019). Low dose of IL-2 combined with rapamycin restores and maintains the long-term balance of Th17/Treg cells in refractory SLE patients. BMC Immunol. *20*, 32.
15. Kimura, K., Hohjoh, H., Fukuoka, M., Sato, W., Oki, S., Tomi, C., Yamaguchi, H., Kondo, T., Takahashi, R., and Yamamura, T. (2018). Circulating exosomes suppress the induction of regulatory T cells via let-7i in multiple sclerosis. Nat. Commun. *9*, 17.
16. Takeuchi, Y., Hirota, K., and Sakaguchi, S. (2020). Impaired T cell receptor signaling and development of T cell–mediated autoimmune arthritis. Immunol. Rev. *294*, 164–176.
17. Yang, L., Li, B., Dang, E., Jin, L., Fan, X., and Wang, G. (2016). Impaired function of regulatory T cells in patients with psoriasis is mediated by phosphorylation of STAT3. J. Dermatol. Sci. *81*, 85–92.
18. Li, B., Lei, J., Yang, L., Gao, C., Dang, E., Cao, T., Xue, K., Zhuang, Y., Shao, S., Zhi, D., et al. (2019). Dysregulation of Akt-FOXO1 Pathway Leads to Dysfunction of Regulatory T Cells in Patients with Psoriasis. J. Invest. Dermatol. *139*, 2098–2107.
19. Moldovan, L.-I., Hansen, T.B., Venø, M.T., Okholm, T.L.H., Andersen, T.L., Hager, H., Iversen, L., Kjems, J., Johansen, C., and Kristensen, L.S. (2019). High-throughput RNA sequencing from paired lesional- and non-lesional skin reveals major alterations in the psoriasis circRNAome. BMC Med. Genomics *12*, 174.

20. Liu, R., Chang, W., Li, J., Cheng, Y., Dang, E., Yang, X., Wang, Q., Wang, G., Li, X., and Zhang, K. (2019). Mesenchymal stem cells in psoriatic lesions affect the skin microenvironment through circular RNA. Exp. Dermatol. *28*, 292–299.

21. Liu, R., Wang, Q., Chang, W., Zhou, L., Li, J., and Zhang, K. (2019). Characterisation of the circular RNA landscape in mesenchymal stem cells from psoriatic skin lesions. Eur. J. Dermatol. *29*, 29–38.

22. Goodman, W.A., Levine, A.D., Massari, J.V., Sugiyama, H., McCormick, T.S., and Cooper, K.D. (2009). IL-6 signaling in psoriasis prevents immune suppression by regulatory T cells. J. Immunol. *183*, 3170–3176.

23. Schjenken, J.E., Moldenhauer, L.M., Zhang, B., Care, A.S., Groome, H.M., Chan, H.-Y., Hope, C.M., Barry, S.C., and Robertson, S.A. (2020). MicroRNA miR-155 is required for expansion of regulatory T cells to mediate robust pregnancy tolerance in mice. Mucosal Immunol. *13*, 609–625.

24. Sánchez-Díaz, R., Blanco-Dominguez, R., Lasarte, S., Tsilingiri, K., Martín-Gayo, E., Linillos-Pradillo, B., de la Fuente, H., Sánchez-Madrid, F., Nakagawa, R., Toribio, M.L., and Martín, P. (2017). Thymus-Derived Regulatory T Cell Development Is Regulated by C-Type Lectin-Mediated BIC/MicroRNA 155 Expression. Mol. Cell. Biol. *37*, e00341-16.

25. Hippen, K.L., Loschi, M., Nicholls, J., MacDonald, K.P.A., and Blazar, B.R. (2018). Effects of MicroRNA on Regulatory T Cells and Implications for Adoptive Cellular Therapy to Ameliorate Graft-versus-Host Disease. Front. Immunol. *9*, 57.

26. Zhu, L., Song, H., Zhang, L., and Meng, H. (2019). Characterization of IL-17-producing Treg cells in type 2 diabetes patients. Immunol. Res. *67*, 443–449.

27. Hovhannisyan, Z., Treatman, J., Littman, D.R., and Mayer, L. (2011). Characterization of interleukin-17-producing regulatory T cells in inflamed intestinal mucosa from patients with inflammatory bowel diseases. Gastroenterology *140*, 957–965.

28. Massoud, A.H., Charbonnier, L.-M., Lopez, D., Pellegrini, M., Phipatanakul, W., and Chatila, T.A. (2016). An asthma-associated IL4R variant exacerbates airway inflammation by promoting conversion of regulatory T cells to TH17-like cells. Nat. Med. *22*, 1013–1022.

29. Bovenschen, H.J., van de Kerkhof, P.C., van Erp, P.E., Woestenenk, R., Joosten, I., and Koenen, H.J.P.M. (2011). Foxp3+ regulatory T cells of psoriasis patients easily differentiate into IL-17A-producing cells and are found in lesional skin. J. Invest. Dermatol. *131*, 1853–1860.

30. Kannan, A.K., Su, Z., Gauvin, D.M., Paulsboe, S.E., Duggan, R., Lasko, L.M., Honore, P., Kort, M.E., McGaraughty, S.P., Scott, V.E., and Gauld, S.B. (2019). IL-23 induces regulatory T cell plasticity with implications for inflammatory skin diseases. Sci. Rep. *9*, 17675.

31. Miyabe, C., Miyabe, Y., Strle, K., Kim, N.D., Stone, J.H., Luster, A.D., and Unizony, S. (2017). An expanded population of pathogenic regulatory T cells in giant cell arteritis is abrogated by IL-6 blockade therapy. Ann. Rheum. Dis. *76*, 898–905.

32. Hwang, S.-M., Sharma, G., Verma, R., Byun, S., Rudra, D., and Im, S.-H. (2018). Inflammation-induced Id2 promotes plasticity in regulatory T cells. Nat. Commun. *9*, 4736.

OMTN, Volume *21*

Supplemental Information

hsa_circ_0003738 Inhibits the Suppressive Function of Tregs by Targeting miR-562/IL-17A and miR-490-5p/IFN-γ Signaling Pathway

Luting Yang, Chen Zhang, Xiaocui Bai, Chunying Xiao, Erle Dang, and Gang Wang

Supplementary Materials and Methods

Circular RNA hsa_circ_0003738 inhibits the suppressive function of Tregs in psoriatic patients by targeting miR-562/IL-17A and miR-490-5p/IFN-γ signaling pathway

Table S1 Upregulated circRNAs in psoriatic Tregs

**P-value**	**FC (abs)**	**circRNA**	**Alias**	**circRNA_ type**
0.034084828	3.3505229	hsa_circRNA_001175	hsa_circ_0001658	exonic
0.030387321	1.5121903	hsa_circRNA_100119	hsa_circ_0002909	exonic
0.032145082	1.5124685	hsa_circRNA_100120	hsa_circ_0000038	exonic
0.040528595	1.7819617	hsa_circRNA_100750	hsa_circ_0020929	exonic
0.04883818	1.6535833	hsa_circRNA_101031	hsa_circ_0007478	exonic
0.044809406	1.547614	hsa_circRNA_101141	hsa_circ_0005785	exonic
0.0458853	1.5419384	hsa_circRNA_101550	hsa_circ_0035796	exonic
0.032128144	1.5279761	hsa_circRNA_101740	hsa_circ_0008616	exonic
0.049057817	2.3222936	hsa_circRNA_101748	hsa_circ_0003645	exonic
0.01810066	2.1755129	hsa_circRNA_101838	hsa_circ_0039930	exonic
0.005943973	1.5254679	hsa_circRNA_103417	hsa_circ_0004788	exonic
0.036592273	1.7508295	hsa_circRNA_103572	hsa_circ_0008351	exonic
0.005949669	4.6603878	hsa_circRNA_104086	hsa_circ_0003738	exonic
0.045574811	1.79207	hsa_circRNA_104499	hsa_circ_0082564	exonic
0.005987429	1.6234627	hsa_circRNA_104940	hsa_circ_0089153	exonic
0.022270222	1.7316345	hsa_circRNA_400091	hsa_circ_0092310	intronic

Table S2 Downregulated circRNAs in psoriatic Tregs

**P-value**	**FC (abs)**	**circRNA**	**Alias**	**circRNA_type**
0.03977296	3.054981	hsa_circRNA_000167	hsa_circ_0000518	intragenic
0.043291906	1.9914322	hsa_circRNA_000274	hsa_circ_0000919	intronic
0.043480055	2.0152613	hsa_circRNA_001059	hsa_circ_0000554	intragenic
0.039331832	1.9960006	hsa_circRNA_001379	hsa_circ_0000516	antisense
0.049249289	2.5803992	hsa_circRNA_001678	hsa_circ_0000517	intragenic
0.043876847	3.0505549	hsa_circRNA_001846	hsa_circ_0000520	intragenic
0.013501149	1.5123283	hsa_circRNA_101592	hsa_circ_0036287	exonic

Table S3 Primers used in this study

Gene symbol		Sequences
Human	Forward	GAGTGTGGAGACCATCAAGGA
IFN-γ	Reverse	GTATTGCTTTGCGTTGGACA
Human	Forward	CGGACTGTGATGGTCAACCTGAAC
IL-17	Reverse	GGTCCTCATTGCGGTGGAGATTC
Human	Forward	AGATACCCTCACCTACACCTTG
RORγT	Reverse	CCGCTCAGGGCTGTATTCAA
Human	Forward	CGTCCTCGTGGAAGTGACAT
RPLP0	Reverse	CATTCCCCCGGATATGAGGC
Human	Forward	TGAGGTGCTGGCTCACATAG

Circ_0003738	Reverse	AACGACGCCATGAATCTCCA
Human	Forward	TCATCGAGCCCATTATGAAACTGA
Circ_00039930	Reverse	AATGAGCTGGTAAAGGCCAGT
Human	Forward	GCCCAATCTCTCCTGCAAGT
Circ_0001658	Reverse	CCACCTAGGAGGAACTGACAA
